# ISCOM-like Nanoparticles Formulated with *Quillaja brasiliensis* Saponins Are Promising Adjuvants for Seasonal Influenza Vaccines

**DOI:** 10.3390/vaccines9111350

**Published:** 2021-11-18

**Authors:** Mariana Rivera-Patron, María Moreno, Mariana Baz, Paulo M. Roehe, Samuel P. Cibulski, Fernando Silveira

**Affiliations:** 1 Departamento de Desarrollo Biotecnológico, Instituto de Higiene, Facultad de Medicina, Universidad de la República (UdelaR), Montevideo 11600, Uruguay; mrivera@higiene.edu.uy (M.R.-P.); mmoreno@higiene.edu.uy (M.M.); 2 Research Center in Infectious Diseases of the CHU of Québec and Université Laval, Quebec City, QC G1V 4G2, Canada; Mariana.Baz@crchudequebec.ulaval.ca; 3 Departamento de Microbiologia Imunologia e Parasitologia, Laboratório de Virologia, UFRGS, Porto Alegre 90050-170, Brazil; proehe@gmail.com; 4 Laboratório de Biotecnologia Celular e Molecular, Centro de Biotecnologia—CBiotec, Universidade Federal da Paraíba, Cidade Universitária, João Pessoa 58051-900, Brazil

**Keywords:** adjuvants, *Quillaja brasiliensis*, ISCOMs, influenza virus, dose-sparing effect

## Abstract

Vaccination is the most effective public health intervention to prevent influenza infections, which are responsible for an important burden of respiratory illnesses and deaths each year. Currently, licensed influenza vaccines are mostly split inactivated, although in order to achieve higher efficacy rates, some influenza vaccines contain adjuvants. Although split-inactivated vaccines induce mostly humoral responses, tailoring mucosal and cellular immune responses is crucial for preventing influenza infections. *Quillaja brasiliensis* saponin-based adjuvants, including ISCOM-like nanoparticles formulated with the QB-90 saponin fraction (IQB90), have been studied in preclinical models for more than a decade and have been demonstrated to induce strong humoral and cellular immune responses towards several viral antigens. Herein, we demonstrate that a split-inactivated IQB90 adjuvanted influenza vaccine triggered a protective immune response, stronger than that induced by a commercial unadjuvanted vaccine, when applied either by the subcutaneous or the intranasal route. Moreover, we reveal that this novel adjuvant confers up to a ten-fold dose-sparing effect, which could be crucial for pandemic preparedness. Last but not least, we assessed the role of caspase-1/11 in the generation of the immune response triggered by the IQB90 adjuvanted influenza vaccine in a mouse model and found that the cellular-mediated immune response triggered by the IQB90-Flu relies, at least in part, on a mechanism involving the casp-1/11 pathway but not the humoral response elicited by this formulation.

## 1. Introduction

Influenza type A and B virus cause respiratory infections in many species, including humans, and the World Health Organization (WHO) estimates that they cause 3–5 million severe cases and 290,000 to 650,000 deaths worldwide yearly [1]. Vaccination is the best cost-effective strategy for preventing infectious diseases including influenza. Currently, licensed influenza vaccines contain either subunit, inactivated, or live attenuated influenza viruses. The most widely used vaccines are subunits containing partially purified surface antigens such as hemagglutinin (HA) and neuraminidase (NA) or split-inactivated vaccines [2]. However, due to the antigenic drifts, shifts, and reassortments that are frequent events with influenza viruses, vaccines must be updated seasonally. Antibodies are crucial for achieving protection from influenza infections and target mostly HA and NA influenza surface antigens [3]. Antibodies targeting the HA head domain are typically neutralizing, as they block the entrance of the virus to the host cell. The main entry pathway for influenza virus are the mucosal surfaces of the respiratory tract; thus, secretory IgA (sIgA) antibodies play a key role in the prevention of the infection, inhibiting viral transmission [4]. In order to elicit adequate immune responses and to achieve higher efficacy rates in specific populations, such as the elderly, some influenza vaccines contain adjuvants [5]. Adjuvants can not only potentiate the immune response of a given vaccine but also provide a dose-sparing effect, polarize, or even accelerate the immune response prompted by the vaccine [6]. Several adjuvants have been licensed in influenza vaccines formulations such as aluminum salts, MF59^®®^, AS03, and AF03 [5]. *Quillaja saponaria* bark-extracted triterpenoid saponins have been widely used as vaccine adjuvants for veterinary vaccines for many decades [7,8]. Recently, a malaria and a herpes zoster vaccine containing the purified saponin QS-21 have been licensed for human use. These are the RTS,S vaccine (Mosquirix^TM^), the first EMA approved vaccine against malaria for endemic population, and Shingrix^®®^, a herpes zoster vaccine for the elderly [9]. In addition, among the different vaccine candidates that are being developed in order to tackle the COVID-19 pandemic, Novavax^®®^ has developed a nanoparticle saponin-based adjuvanted vaccine, named NVX-CoV2373, for which a Th1 biased immune response was detected during phase I and II [10], and it has shown an efficacy of 86.3% against the B.1.1.7 (alpha) variant and 96.4% against non-B.1.1.7 variants during phase III [11].

Immune-stimulating complexes (ISCOMs) are cage-like nanoparticles of approximately 40 nm, obtained combining *Q. saponaria* saponins, lipids, and protein antigens [12]. Our group has been studying the adjuvant capacity of saponins obtained from the leaves of *Q. brasiliensis* [13,14,15,16,17,18,19], a native tree from South America [20]. Previous reports have shown remarkable similarities between QB-90 fraction and QuilA saponins, both in their chemical structure and from an immunological properties perspective when used with herpesvirus [21] and poliovirus [14] antigens in mouse models. In addition, we have previously reported that QB-90 is less toxic than QuilA both in vivo and in vitro, suggesting that QB-90 could be used as an alternative to QuilA [16,22]. Furthermore, using the model antigen ovoalbumin (OVA), we reported for the first time that soluble saponins, either QuilA or QB-90, are able to trigger both humoral and cellular immune response in a similar manner. Likewise, the ISCOM nanoparticles formulated with them, either QB-90 (IQB90) or QuilA (IQuilA), induce comparable immune responses both at humoral and cellular levels [22]. In particular, we demonstrated that IQB90 improves antigen uptake, induces a potent cellular-mediated immune response (CMI) and induces a strong humoral immune response, both at mucosal and systemic levels, in a mice model [22]. Recently, we reported that matrices of IQB90 (ISCOMs without antigen) are able to trigger early local and systemic cellular responses [23]. In addition, we reported that this formulation is able to activate inflammasome complexes through caspase-1/11 and MyD88 pathways in vitro. Moreover, the IQB90 formulation was proven to induce a strong mixed Th1/Th2 adaptive immune response [23]. Herein, we demonstrate that a split-inactivated IQB90-adjuvanted influenza vaccine triggers a stronger immune response than a commercial unadjuvanted vaccine, when applied either by the subcutaneous (s/c) or the intranasal (i.n.) route, and that the triggered response is adequate to confer protection against an influenza infection. In addition, we reveal that this novel adjuvant not only enhances the potency of the immune response but also confers up to a ten-fold dose-sparing effect. Finally, we assessed the role of caspase-1/11 in the generation of the adaptive immunity in response to vaccination with an IQB90 adjuvanted-influenza vaccine in mice.

## 2. Materials and Methods

### 2.1. Vaccine Adjuvants

Leaves from adult plants of *Q. brasiliensis* (A. St.-Hil. et Tul) Mart. were collected in Canguçu, RS, Brazil (31°23′42” S–52°40′32” W) (voucher ICN 142953, deposited at the Herbarium of the Federal University of Rio Grande do Sul). Extraction and purification of QB-90 saponin fractions were carried out as previously described [24].

### 2.2. Antigen Preparation

Antigen was obtained from trivalent split-inactivated influenza virus vaccine (Instituto Butantan, *Myxovirus influenzae* 2016 strains A/California/7/2009 (H1N1) pdm09, A/Hong Kong/4891/2014 (H3N2), B/Brisbane/60/2008 (Victoria lineage) south hemisphere, batch N3B82, Ministério da Saúde, Governo Federal, Brazil) containing 15 µg of hemagglutinin (HA) per strain in 0.5 mL. Briefly, 24.5 mL of the antigen suspension were concentrated using Amicon^®®^ Ultra-15 30K (Millipore, Ref: UFC 903008). Antigen concentration was calculated considering the initial concentration of hemagglutinin (90 µg/mL), the initial (24.5 mL) and the final volume (1.593 mL) obtained with a retention rate of 10%, according to manufacturer’s instructions. The desired final concentration (1 mg/mL of HA per influenza strain) was attained by adding an appropriate volume of saline solution.

### 2.3. Experimental Vaccine Preparations

#### 2.3.1. Unadjuvanted Influenza Vaccine

The 1 mg/mL antigen solution obtained previously was diluted in saline solution in order to obtain a final concentration of 25 µg/mL of HA per influenza strain (total HA content 75 µg/mL).

#### 2.3.2. IQB90 Adjuvanted Influenza Vaccine

ISCOMs with viral antigen (IQB90-Flu) were prepared by the modified ethanol injection technique [22,25]. Briefly, the concentrated viral antigen solution (1 mg/mL in PBS, pH 7.4) was added to a solution of *Q. brasiliensis* saponins (QB-90, 1 mg/mL). Ethanol-dissolved cholesterol (Sigma, Saint Louis, MO, USA) and di-palmitoyl phosphatidylcholine (Avanti Polar Lipids, Birmingham, AL, USA) were immediately injected into the mixture, which was gently stirred for 48 h at 4 °C. After that procedure, ISCOMs derived from QB-90 fraction containing the influenza viral antigen (IQB90-Flu) were obtained. The final concentration of QB-90 saponins used in the formulation of the IQB90-Flu was 50 µg/mL. Three formulations of IQB90-Flu were prepared with different concentrations of total HA antigen (75, 7.5, or 0.75 µg/mL). All three formulations contained 50 µg/mL of QB-90 within the ISCOMs. Nanoparticle assembly was confirmed visually by transmission electron microscopy (TEM). In order to determine the toxicity of the IQB90-Flu with the three preparations, hemolysis assays were performed as previously described [22].

### 2.4. Transmission Electron Microscopy (TEM)

An aliquot (10 µL) of an aqueous solution of IQB90-Flu was placed on a copper grid covered with formvar and carbon film (200 mesh) and negatively stained with 2% phosphotungstic acid (pH 7.2) for 2 min at room temperature. Once dried, the samples were examined with a JEM 1010 transmission electron microscope (JEOL, Japan), operated at an 80 kV accelerating voltage and at magnifications between 30,000 and 150,000×.

### 2.5. Mouse Immunizations and Sample Collection

Animal manipulation was performed in accordance with Comisión Honoraria de Experimentación Animal (CHEA) guidelines and was approved by the Ethics Committee of Universidad de la República (CHEA, Udelar, CHEA approval protocol nº 468).

Casp-1/11 knockout (ko) and littermate (wt) female C57Bl/6 mice (8–12 weeks old) were purchased from the Institut Pasteur Montevideo (Uruguay), and female BALB/c mice (8–12 weeks old) were purchased from DILAVE (MGAP-Uruguay). All animals were kept at the Instituto de Higiene (Montevideo, UdelaR) animal breeding facility, housed in ventilated racks under controlled temperature (22 ± 2 °C) and humidity (50–60%) in a 12/12 h light/dark cycle, with food and water ad libitum. Prior to the beginning of the experiments, animals were acclimatized over one week under the controlled conditions previously indicated.

Female BALB/c mice were immunized on days 0 and 14 with previously described formulations containing viral antigen through subcutaneous (s/c) or intranasal (i.n.) routes. S/c immunizations (*n* = 9) were performed with 100 µL in the hind neck with 75, 7.5, or 0.75 µg/mL of total HA (antigen) contained in IQB90-Flu or 75 µg/mL of antigen alone (Flu). For intranasal immunization, mice (*n* = 10) were anesthetized with ketamine–xylazine and received 100 µL of 75 µg/mL of antigen per dose in vaccinated groups, either IQB90-Flu or alone (unadjuvanted). Bleedings were performed immediately prior to inoculations (days 0 and 14) and 2 weeks after the second immunization (day 28). Sera were stored at −20 °C until use.

C57Bl/6 casp-1/11 ko and wt female mice (*n* = 5) were immunized on days 0 and 14 through the s/c route in the hind neck with 100 µL of previously described formulations containing 75 µg/mL of total HA (antigen) either in IQB90-Flu, without adjuvant (Flu), or with saline solution (without antigen nor any adjuvant) as control. Bleedings were performed immediately prior to inoculations (days 0 and 14) and 2 weeks after the second immunization (day 28). Sera were stored at −20 °C until use.

### 2.6. Delayed-Type Hypersensitivity (DTH) Assay

Delayed-type hypersensitivity (DTH) responses were evaluated as described previously [13]. Briefly, casp-1/11 wt and ko C57Bl/6 mice previously inoculated twice with adjuvanted formulation (IQB90-Flu, *n* = 5), antigen alone (Flu, *n* = 5), or with physiological solution only (*n* = 3) received one intradermal (ID) injection of 10 µL containing 0.075 µg of total HA in right hind footpad. The thickness of the injected (right) and non-injected (left) footpads were measured 24 and 48 h later with a caliper. The swelling of mice from the control group (previously inoculated only with physiological solution) was considered as basal swelling. The vaccine-induced influenza-specific DTH response of each animal was calculated based on the thickness of its injected footpad minus the thickness of its non-injected (left) footpad.

### 2.7. Antibody-Level Determination Assays

The antibodies against the influenza virus (i.e., IgG total, IgG1, IgG2b, IgG2a, IgG3, and IgGA) were assayed by indirect ELISA as described previously [22]. Briefly, 96-well ELISA plates (Greiner Bio-One, Darmstadt, Germany) were coated overnight at 4 °C with the vaccine influenza antigen described above (1.0 µg/mL) diluted in PBS (pH 7.2). Plates were then washed three times with PBS containing 0.05% Tween^®®^ 20 (Sigma, Saint Louis, MO, USA) (PBST) and blocked with 1% Tween^®®^ 20 in PBS at 37 °C for 2 h. Appropriately diluted mouse serum samples were added in duplicate (100 µL/well) and incubated for 1 h at 37 °C. After washing three times with PBST, HRP-conjugated anti-mouse with either total IgG (cat. nº 1030-05 Southern Biotech Birmingham, AL, USA), IgG1 (cat. nº 1071-05 Southern Biotech Birmingham, AL, USA), IgG2b (cat. nº 1091-05 Southern Biotech Birmingham, AL, USA), IgG2a (cat. nº 1081-05 Southern Biotech Birmingham, AL, USA), IgG3 (cat. nº 1101-05 Southern Biotech Birmingham, AL, USA), or IgG2c (cat. nº 1079-05 Southern Biotech Birmingham, AL, USA) diluted 1/10,000 in PBST were added to each well, and plates were incubated for 1 h at 37 °C. After three washings, 100 µL of ortho-phenylenediamine (OPD; Sigma, Saint Louis, MO, USA) with 0.003% H_2_O_2_ were added, and plates were incubated for 30 min at 25 °C. Reactions were stopped with 30 µL/well of 1N HCl. Optical densities (ODs) were measured in an ELISA plate reader (Sunrise, Tecan Group Ltd., Männedorf, Switzerland) at 490 nm. A standard curve was built using a pool of positive sera, and antibody levels were expressed in arbitrary units per mL (AU/mL).

The hemagglutination inhibition (HAI) titer was determined by measuring the serum inhibition of agglutination by vaccine-derived HA and turkey red blood cells (RBCs). Serum samples were treated with RDE enzyme (receptor-destroying enzyme from *Vibrio cholerae*, Sigma–Aldrich, Saint Louis, MO, USA) and then were tested for HAI activity against the trivalent fragmented and inactivated influenza virus vaccine (Instituto Butantan, Ministério da Saúde, Governo Federal, Sao Paulo, Brazil) using turkey red blood cells. The HAI titer was expressed as the dilution of sample that inhibited influenza virus agglutination of RBC.

### 2.8. ELISpot Assay for Influenza-Specific IgG Antibody Secreting Cells

On day 30 post-priming, mice were euthanized by cervical dislocation, the spleen removed, and splenocytes were obtained aseptically. The frequency of influenza virus-specific IgG antibody-secreting cells (ASCs) was determined using the ELISpot assay as described previously [26]. Briefly, 96-well ELISpot plates (Millipore, Darmstadt, Germany) were wetted with a 15 µL/well of 35% ethanol and left to almost dry for several minutes as indicated by the manufacturer. Plates were then washed with filtered phosphate saline buffer (PBS) and coated for 2 h at 37 °C with 7.5 µg/mL of HA influenza antigen. After washing with filtered PBS, plates were blocked with 200 µL of 1% (w/v) bovine serum albumin (BSA, Sigma, Saint Louis, MO, USA) overnight (ON) at 4 °C. Plates were washed again and 2 × 10^5^ splenocytes (in 100 µL of RPMI 1640 (Gibco) supplemented with 10% fetal calf serum) from vaccinated mice 30 days post-priming were added and incubated overnight at 37 °C under a 5% CO_2_ atmosphere. After incubation, cells were removed by washing, and horseradish peroxidase (HRP)/goat anti-mouse total IgG conjugate (cat. nº 1030-05 Southern Biotech Birmingham, AL, USA) was added and incubated for 2 h at 37 °C. Plates were washed and stained with aminoethylcarbazol (AEC) solution in acetate buffer and 0.1% hydrogen peroxide for 30 min protected from light at room temperature. The reaction was stopped by thorough rinsing with tap water, and plates were dried at room temperature for 3 days. Spots were counted and the result obtained is expressed as the number of anti-influenza IgG ASC per 2 × 10^5^ cells.

### 2.9. Mouse Challenge

Madin–Darby canine kidney (MDCK) cells (ATCC CCL-34) were cultured in EMEM supplemented with 10% of fetal bovine serum (SFB) and the antibiotics penicillin and streptomycin (Gibco) at 37 °C/5% CO_2_. When cells reached ~80% confluence, 2009 H1N1 pandemic influenza virus (A/Brazil/G3P1/2013 (H1N1; GenBank accession number: KP027598-KP027605) was inoculated in the presence of N-tosyl-L-phenylalanine chloromethyl ketone (TPCK)-treated trypsin (5 µg/mL). After an adsorption period of 1 h at 37 °C, 15 mL of EMEM with 1 µg/mL TPCK was added to the flasks and the cytopathic effect (CPE) was monitored. When CPE reached ~80%, the supernatants were recovered and titrated in plaque-forming units (PFUs) per mL using standard procedures.

Four weeks (28 days) after immunization of female BALB/c mice, mice (*n* = 6) were anesthetized with ketamine–xylazine and intranasally challenged with a sublethal dose of 10^3^ PFU of (H1N1) pdm2009 in 50 µL. Animals were visually inspected throughout the study challenge and weighed on a daily basis for fourteen days.

### 2.10. Statistical Analyses

Statistical significance was assessed by Kruskal–Wallis with uncorrected Dunn’s post-test to compare among different groups of mice. When only two groups were compared, a non-parametric *t*-test and Mann–Whitney post-test were used. In the case of loss of weight, a two-way ANOVA (with matched values stacked into a sub-column) analysis was performed, and Fisher’s LSD post-test to compare among different groups of mice (considering unadjuvanted inoculated group as the control group, unless indicated differently). Statistically significant differences were considered when *p* ≤ 0.05. In all cases, GraphPad Prism version 8.4.2 (679) software (GraphPad Software, LCC) was used.

## 3. Results and Discussion

### 3.1. QB-90 ISCOMs Formulated with Influenza Antigen (IQB90-Flu) Are Non-Toxic Cage-Like Structures

**Figure 1:**
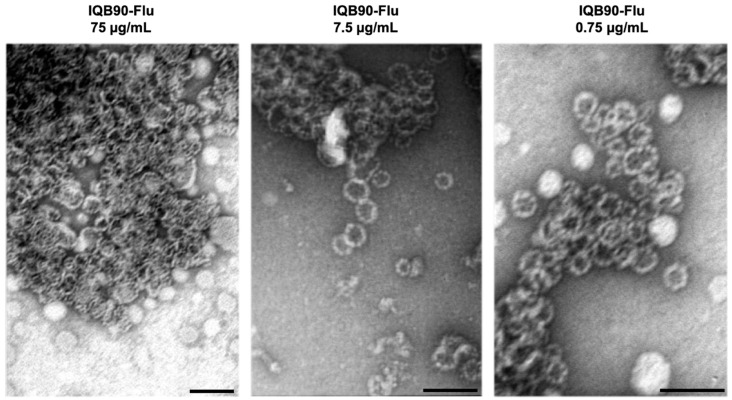
**Transmission electron microscopy** (**TEM**) **of ISCOMs of QB-90 saponins and influenza antigen** (**IQB90-Flu**)**.** ISCOMs were produced by the ethanol injection method with QB-90 saponins and influenza antigen as previously described. Preparations were stained with 2% phosphotungstic acid and observed by TEM. Scale bars represent 100 nm. IQB90-Flu appeared as cage-like nanoparticles of approximately 40 nm.

The QB-90 ISCOMs co-formulated with influenza antigen (IQB90-Flu) were visualized by transmission electron microscopy (TEM), showing the expected cage-like structures of approximately 40 nm (Figure 1).

The hemolysis of red blood cells is a major indicator of the toxicity of saponins and must be evaluated when the development of saponin-based adjuvants is the objective. As previously reported, ISCOM nanoparticles impair the hemolytic capacity of saponins [22]. All IQB90-Flu formulations have been shown not to cause membrane damage according to the results of a hemolysis assay (data not shown). Moreover, IQB90 have been previously proven to be safe and well tolerated in mice in terms of toxicity [22,23]; here, again, the experimentally vaccinated animals in this study did not show any signs of distress (i.e., lethality, local swelling, loss of hair, and piloerection) after the administration of two doses of the adjuvanted split-inactivated seasonal influenza vaccine. In addition, commercial preparations of ISCOM-based adjuvants (derived from *Q. saponaria* saponins) have been demonstrated to be safe and well tolerated in mice, other animals [27], and humans [28,29,30].

### 3.2. Intranasal or Subcutaneous Administration of IQB90-Flu Vaccine Induced Higher Antibody Levels Than the Commercial Influenza Vaccine in Mice

**Figure 2:**
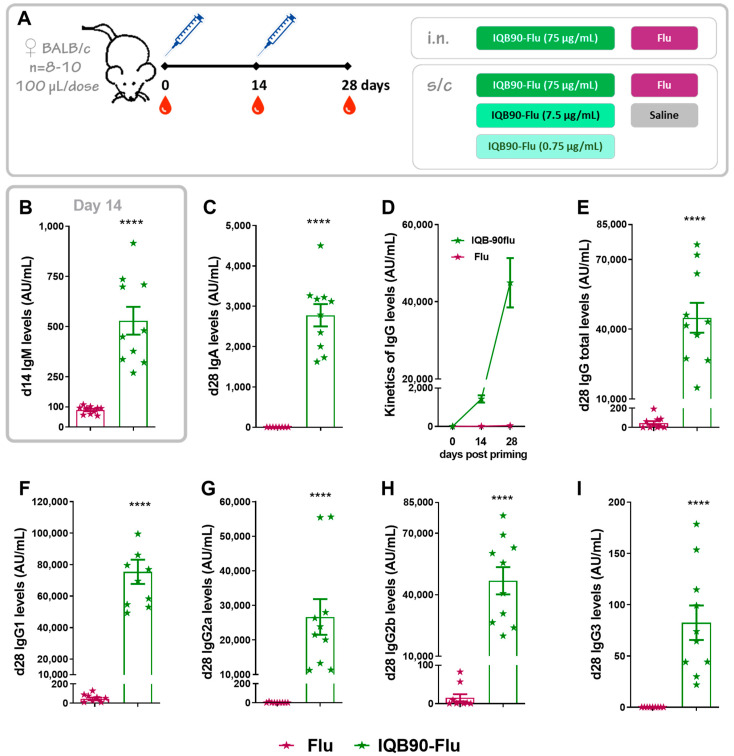
**Intranasal administration of IQB90-Flu adjuvanted influenza vaccine elicited higher antibody levels in sera than the commercial vaccine**. Schematic vaccination schedule (**A**). Immunizations were performed intranasally at day 0 (priming) and 14 (booster) either with an IQB90 adjuvanted formulation (“IQB90-Flu”, green stars) or with no adjuvant (“Flu”, commercial vaccine, pink stars), and antibody levels were measured in sera. Specific anti-influenza IgM levels 14 days post-priming are shown in (**B**), and IgA antibody levels 28 days post-priming are shown in (**C**). Total IgG kinetics are shown in (**D**), and total IgG (**E**), IgG1 (**F**), IgG2a (**G**), IgG2c (**H**), and IgG3 (**I**) antibody levels against influenza antigen were measured in sera 28 days post booster. **** Indicates *p* < 0.0001, Mann–Whitney’s test.

In order to determine the humoral immune response elicited by the formulation, BALB/c mice were inoculated as shown in Figure 2A. The IQB90-Flu delivered intranasally was able to elicit higher levels of IgM antibody levels 14 days post-priming (Figure 2B) and higher IgA antibody levels 28 days post-priming (Figure 2C), higher than Flu. In addition, the IQB90-Flu vaccine induced higher total IgG antibody levels than Flu throughout the study (Figure 2D), in particular, 28 days post-priming (Figure 2E). Regarding the isotypes of the antibodies elicited at day 28 post-priming, the IBQB90-Flu vaccine was able to elicit higher IgG1 (Figure 2F), IgG2a (Figure 2G), IgG2b (Figure 2H), and IgG3 (Figure 2I) antibody levels than the unadjuvanted vaccine.

**Figure 3:**
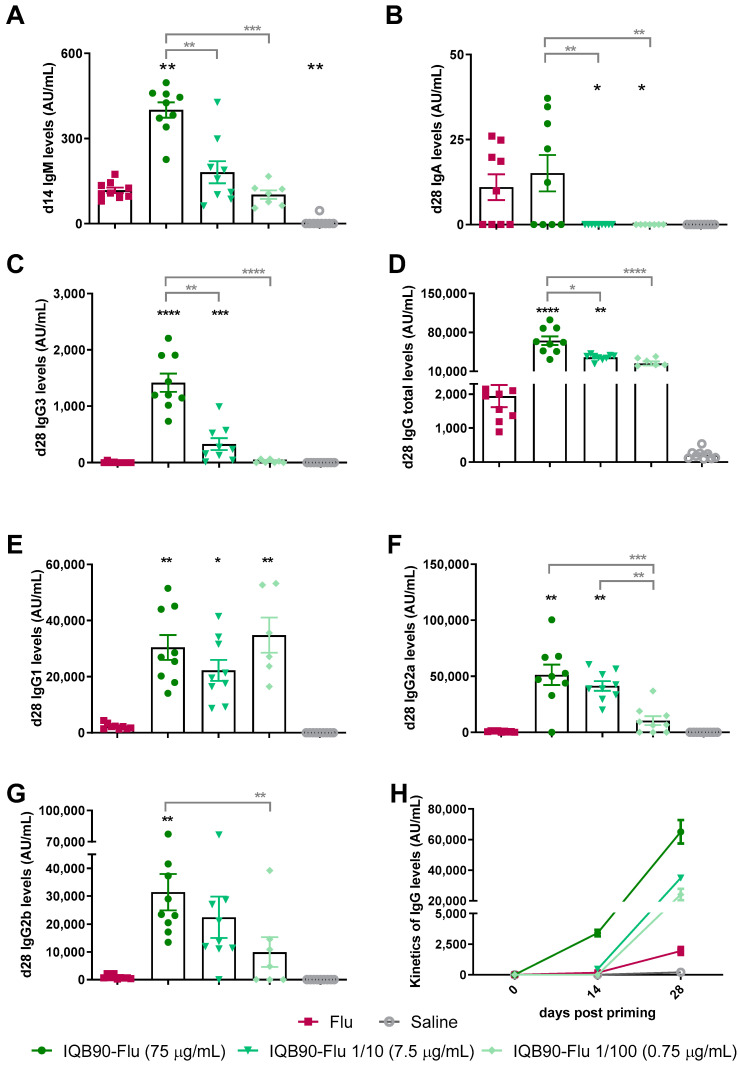
**Subcutaneous administration of IQB90-Flu elicited higher antibody levels than the commercial vaccine and pro-moted a dose-sparing effect**. Immunizations were performed subcutaneously at day 0 (priming) and 14 (booster) either with adjuvanted formulation or with no adjuvant. Specific anti-influenza IgM antibody levels 14 days post-priming are shown in (**A**), and sera IgA antibody levels 28 days post-priming are shown in (**B**). Total IgG (**C**), IgG1 (**D**), IgG2a (**E**), IgG2c (**F**), and IgG3 (**G**) antibodies against influenza antigen were measured in sera 28 days after priming. The kinetics of Total IgG levels is shown in (**H**). To assess the dose-sparing effect conferred by the IQB90 adjuvant, two groups were inoculated with 1/10 (IQB90-Flu 7.5 µg/mL) and 1/100 (IQB90-Flu 0.75 µg/mL) of the amount of antigen contained in the group inoculated with the unadjuvanted formulation. * *p* < 0.05, ** *p* < 0.01, *** *p* < 0.001, and **** *p* < 0.0001, Kruskal–Wallis and Dunn’s post-test (every group against the Flu group are indicated by the black stars, and the adjuvanted groups compared among each other are indicated by grey stars).

When mice were vaccinated following the same schedule via the subcutaneous (s/c) route, IQB90-Flu (75 μg/mL) vaccinated mice elicited a higher total IgM antibodies level than mice inoculated with the non-adjuvanted vaccine (Figure 3A). At day 28 post-priming, total IgA (Figure 3B), IgG (Figure 3C), IgG1 (Figure 3D), IgG2a (Figure 3E), IgG2b (Figure 3F), and IgG3 (Figure 3G) levels were higher in groups inoculated with the adjuvanted vaccines than in mice inoculated with the commercial Flu vaccine. The kinetics of total IgG at days 0, 14, and 28 is shown in Figure 3H.

The induction of mucosal immunity would contribute to generating specific secretory IgA antibodies that would be expected to prevent the infection of a respiratory pathogen and promote herd immunity [31], while the IgG systemic response woud be expected to lead to more effective protection [32]. In addition, the i.n. route is easily accessible and the mucosal tissues are highly vascularized; thus, an immune response can be triggered at distant mucosal sites (due to the dissemination of effector immune cells in the common mucosal immune system), and last but not least, as the mucosal immunization does not require the use of needles, it prevents potential sources of infection with other agents [33]. On the other hand, to prompt an efficient immune response in the mucosal airway is a challenging task due to the tolerogenic mechanisms that underlie the mucosal tissues. The antigen uptake and presentation in the mucosal airway tends to be more complex than in other tissues. For these reasons, the adjuvants to be used in mucosal immunizations must be able to trigger an efficient immune response. It has been reported that to achieve such an aim, adjuvants must promote antigen uptake by mucosal dendritic cells (DCs), key players in triggering adaptive immune responses in the lung, which lead to appropriate antigen presentation and activation of T and B cells. Nonetheless, it has been shown that particulate substances are more properly internalized by the mucosal-associated lymphoid tissue DCs [34]. The shape, size, and nature of the ISCOMs are crucial in order to improve the internalization of the antigen by DCs and avoid phagocytic cells, such as alveolar macrophages [35], as has been reported for *Q. saponaria*-derived ISCOMs in influenza vaccines [36]. In addition, the adjuvant must be safe, well tolerated and, importantly, it must not affect the central nervous system. Unfortunately, previously, a live-attenuated influenza vaccine adjuvanted with *E. coli* heat-labile toxin (LT) had to be withdrawn due to the latter issue [5]. Instead, ISCOMs have been proven to be well tolerated and safe [30]. Taking into account these considerations and the immune response triggered by IQB90-Flu (Figure 2), *Q. brasiliensis*-derived ISCOMs show potential as adjuvants to intranasal influenza vaccines.

### 3.3. IQB90-Flu Conferred a Ten-Fold Dose-Sparing Effect in a Split-Inactivated Influenza Vaccine

As the amount of antigen required per vaccine dose could be an important issue in the preparedness for a new influenza pandemic, which might result in a larger number of doses available to the population, we assessed whether the IQB90-Flu adjuvanted vaccine was able to induce a dose-sparing effect. In previous studies, we have demonstrated that an aqueous extract of *Q. brasiliensis* leaves (AE), when used as a vaccine adjuvant, was able to induce a dose-sparing effect in formulations containing even one-fifth of the original antigen in bovine viral diarrhea virus (BVDV) experimental vaccines [18]. Therefore, in order to assess whether this would be valid for influenza vaccines, two additional groups were inoculated subcutaneously with the adjuvanted experimental vaccine containing 50 µg/mL of QB-90 saponins, and either one-tenth (“IQB90-Flu 1/10” containing 7.5 µg/mL of total HA) or one-cent (“IQB90 1/100” containing 0.75 µg/mL of total HA) of the antigen contained in the “Flu” formulation.

No statistically significant differences among adjuvanted groups were observed at day 28 in the antibody levels of IgG1, IgG2a, and IgG2b (Figure 3) in the groups inoculated with 75 and 7.5 µg/mL of HA in IQB90 adjuvanted formulations (Figure 3F,G), indicating that a dose-sparing effect was induced by the IQB90 adjuvant in these cases. In addition, groups inoculated with 7.5 µg/mL of HA were able to induce higher levels of total IgG, IgG1, IgG2a, and IgG3 than the group inoculated with the unadjuvanted formulation (Figure 3D–F,H). However, statistically significant differences (indicated by black stars) were found in IgA levels, where only full dose of IQB90-Flu adjuvanted vaccine (with 75 µg/mL of HA) did not induce significantly lower levels of IgA in comparison with the unadjuvanted formulation (Figure 3B). In addition, differences were detected between the adjuvanted groups in the total IgG and IgG3 antibody levels elicited, indicating that rather than a dose-sparing effect, a dose-dependent effect was observed (Figure 3D,H). In both cases, the formulations containing higher amounts of antigen were able to elicit significantly higher antibody levels. In addition, the formulation containing one-tenth of HA (7.5 µg/mL) was able to elicit higher IgG2a antibody levels than that containing a hundredth of HA (Figure 3F). Therefore, it is important to bear in mind that the dose-sparing effect was maintained when the amount of antigen was decreased 10-fold.

### 3.4. The Immune Response Elicited by Subcutaneous Injection of IQB90-Flu Vaccines Was Adequate for Conferring Protection against Influenza Infection in Mice

**Figure 4:**
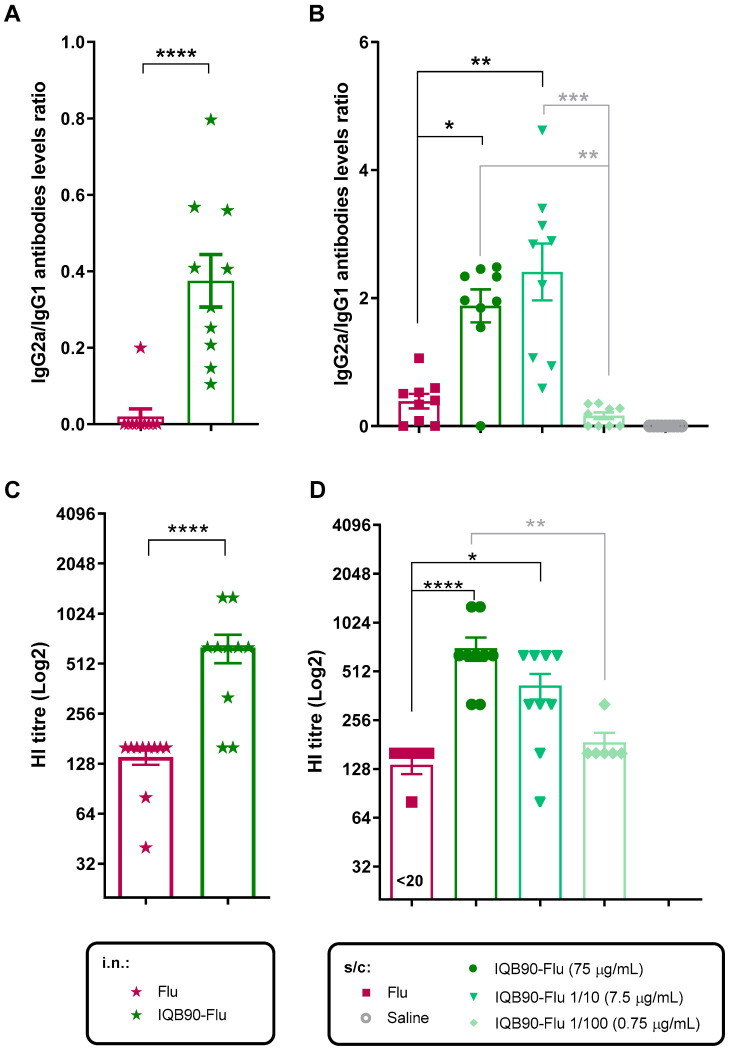
**Functionality of humoral response elicited by IQB90 adjuvanted vaccines.** IgG2a/IgG1 ratios of antibody levels elicited at day 28 by intranasal (**A**) or subcutaneous (**B**) inoculations of IQB90 adjuvanted (green symbols and bars) or unadjuvanted (pink symbols and bars) seasonal influenza vaccines. Antibodies able to inhibit hemagglutination, elicited at day 28 post-priming by i.n. (**C**) or s/c (**D**) administration of the adjuvanted or unadjuvanted influenza vaccines, were assessed by HAI as described in Section 2. Statistical analyses to compare the two groups (**A**,**C**) were performed using the Mann–Whitney test. When three or more groups were compared (**B**,**D**), statistical analyses were performed using the Kruskal–Wallis and uncorrected Dunn’s post-test, comparing every group against the unadjuvanted (Flu) group as the control. Statistically significant differences are considered at * *p* < 0.05, ** *p* < 0.01, *** *p* < 0.001 and **** *p* < 0.0001; black stars indicate Flu, and grey stars are used for comparisons among adjuvanted groups.

Eliciting IgG2a antibody levels is crucial for influenza (as well as many other) vaccines. This isotype has been shown to play a pivotal role in protection. IgG2a antibodies bind to Fc receptors with higher affinity than to other isotypes, activating antibody-dependent cell-mediated cytotoxicity (ADCC) and the opsonophagocytosis mechanisms towards influenza infected cells. When the IgG2a/IgG1 ratio was assessed in the present study, we found that in the case of intranasal immunization, such a ratio was smaller than one (Figure 4A), meaning that higher IgG1 antibody levels were elicited than those of IgG2a. In contrast, the IgG2a/IgG1 ratio elicited by the subcutaneous inoculation of IQB90 adjuvanted vaccine was greater than one (Figure 4B), suggesting that the immune response was polarized towards a Th1 phenotype. This observation is consistent with previous studies by our group in which *Q. brasiliensis* saponin-based adjuvants were used for other viral antigens by subcutaneous route [13,14,15,18] and for other adjuvanted seasonal influenza experimental [37] or licensed vaccines inoculated subcutaneously [38].

In order to determine the ability of the elicited influenza-specific antibodies to inhibit the hemagglutination, we performed a hemagglutinin inhibition (HAI) assay using turkey RBCs as referred to in Section 2. Antibodies that are able to target the head-domain of HA, preventing the binding of the viral HA to the cellular receptor, are inhibitors of hemagglutination and typically neutralizing. However, it is important to recall that HAI titers do not correlate *perfectly* with protection, though HAI is still considered a *surrogate marker* of vaccine efficacy. Nevertheless, the HAI assay constitutes an appropriate tool to address this issue, even at a clinical level, as it is globally accepted by regulatory agencies such as the FDA [3,39]. Moreover, a demonstration of the HAI response is a requisite for licensing influenza vaccines in the USA [40].

In the present study, the HAI titers were higher in IQB90-Flu adjuvanted vaccines than in the unadjuvanted vaccine, when inoculated either by the i.n. (Figure 4C) or by s/c route (Figure 4D). In addition, no statistically significant differences were observed among groups of mice inoculated subcutaneously with the full amount of antigen and those inoculated with one-tenth of the amount of antigen (Figure 4D). This is of remarkable importance, as it implies that the dose-sparing effect conferred by the IQB90-Flu adjuvant observed in the antibody levels (Figure 3) was effective, as the antibodies elicited with one-tenth of the amount of antigen present in the commercial vaccine were able to induce similar levels of significant HAI titers, thus suggesting potential protection to influenza infections.

Overall, these results indicate that the IQB90-Flu formulations, either at 75 or 7.5 µg/mL, were still able to induce important antibody levels and a balanced Th1/Th2 type of immune response, polarized towards the Th1 phenotype when the s/c route was used, which has proven to be effective in conferring protection against seasonal influenza infections [40].

### 3.5. IQB90-Flu Vaccines Trended to Protect Mice from Influenza

**Figure 5:**
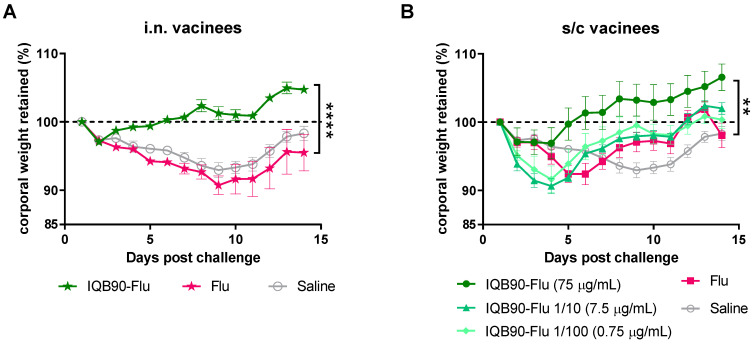
**Protective effect of IQB90-Flu vaccines.** BALB/c mice were vaccinated twice (two weeks apart, on day 0 and 14), by the i.n. (**A**) or s/c (**B**) route. On day 28 post-priming, mice were challenged with a dose of 10^3^ PFU of A(H1N1)pdm2009, by the i.n. route. Animals were observed on a daily basis for clinical signs of disease and survival, and their body weight was measured. Corporal weight changes in percentage (mean and error) are shown, considering as 100% their body weight previous to challenge. Statistically significant differences are indicated by black stars: ** *p* < 0.01 and **** *p* < 0.0001; two-way ANOVA and Fisher’s LSD test, comparing every group against the Flu group.

With the aim of assessing whether the elicited immune response by the IQB90-Flu vaccines was protective against influenza infection, s/c or i.n. vaccinated BALB/c mice were subjected to a sub-lethal challenge with 10^3^ PFU of A(H1N1)pdm2009, delivered intranasally. After challenge, mice were monitored daily over 14 days for clinical signs of disease (i.e., piloerection, curved back, abnormal behavior, and survival) and weight loss. A group of mice inoculated with saline was maintained as the negative control and also subjected to challenge (Figure 5). In addition, sentinel animals (neither vaccinated nor challenged) were included in all groups to ensure appropriate weight controls (not shown).

As shown in Figure 5, animals vaccinated with the full dose of antigen (IQB90-Flu 75 µg/mL), either by the i.n. (Figure 5A) or s/c route (Figure 5B), were either able to keep their body weight at 100% or gained weight throughout the study, despite a slight weight decrease in the first 2–4 days post-challenge. On the other hand, mice inoculated with unadjuvanted vaccine, either by the i.n. (Figure 5A) or s/c route (Figure 5B), had a rapid decrease in body weight, which did not recover until the end of the assay at 14 days post-challenge. Importantly, mice that received IQB90-Flu containing one-tenth or one-cent of the amount of antigen by the s/c route showed a decrease in their body weight in the days following challenge but recovered rapidly.

### 3.6. Subcutaneous Administration of IQB90 Elicited High Antibody Levels and Specific ASC in Wild-Type and Casp-1/11 Knockout Mice

**Figure 6:**
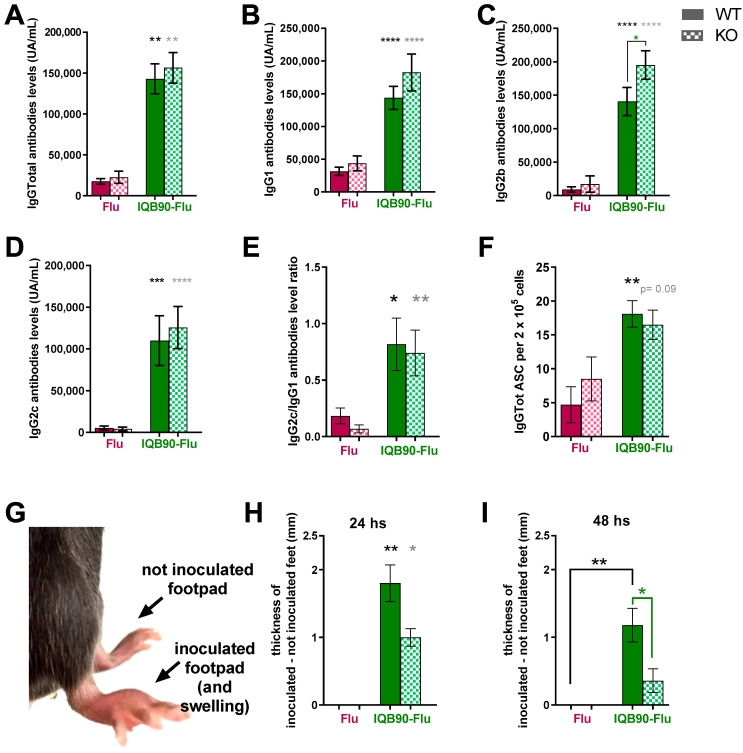
**Humoral and cellular immune responses in wt and Casp-1/11 knockout mice inoculated with IQB90-Flu.** Female C57Bl/6 mice (*n* = 5) were inoculated subcutaneously on days 0 (priming) and 14 (booster) either with IQB90-Flu (green bars) or the unadjuvanted formulation (Flu, pink bars). Two weeks after booster, humoral, and cellular immune responses were assessed. Specific anti-influenza total IgG (**A**), IgG1 (**B**), IgG2b (**C**), and IgG2c (**D**) antibody levels were measured in sera at day 28. The IgG2a/IgG1 ratio of antibody levels are shown in (**E**). At the same time point, splenocytes were obtained aseptically, and specific anti-influenza IgG ASC were assessed by ELISpot (**F**). The results are expressed as the number of ASC every 2 × 10^5^ splenocytes. To study the CMI response, two weeks after booster, mice were injected with 0.75 µg of influenza antigen by the intradermal route at the right hind foot pad. 24 (**H**) and 48 (**I**) hours later, the thickness of both the inoculated (right pad) and non-inoculated (left pad) were measured using an electronic caliper. The swelling was calculated as the thickness of the inoculated minus the thickness of the non-inoculated pad; an example of a wt mouse vaccinated with IQB90 and 24 h post-intradermal inoculation is shown (**G**). Two groups of mice (wt and ko mice, *n* = 3 each) were mock inoculated with saline solution. No swelling was detected after the inoculation of the influenza antigen (the differences between the two footpads were zero for every animal in both the wt and ko groups at 24 and 48 h, data not shown). * *p* < 0.05, ** *p* < 0.01, *** *p* < 0.001, and **** *p* < 0.0001, two-way ANOVA and Fisher’s post-test were used to compare between the wt and ko groups (colored stars), and the Kruskal–Wallis test and uncorrected Dunn’s post-test were used to compare every group against the unadjuvanted (Flu) group as the control (black stars represent wt and grey stars represent ko groups).

Commercial ISCOM-matrices (formulated using *Q. saponaria* saponins) have been reported to activate the NALP-3–ASC–caspase-1 (NLRP3) inflammasome in APCs [41], as well as other particulate adjuvants such as alum [42]. Particularly, *Q. saponaria* saponins activate the NLRP3 inflammasome pathway in LPS-primed macrophages [41]. Previously, we demonstrated that IQB90 induces IL-1β production in bone-marrow dendritic cells (BMDCs) in vitro in a caspase-1/11-dependent manner [23]. We ought to determine whether inflammasome complexes are involved in IQB90-Flu vaccine-induced response in vivo. To achieve this, we inoculated caspase-1/11 knockout (ko) or wild type (wt) in C57Bl/6 background mice with two doses (two weeks apart) of either IQB90-Flu, Flu, or saline solution. As shown in Figure 6, IQB90-Flu was able to elicit higher levels of specific anti-influenza total IgG (Figure 6A), IgG1 (Figure 6B), IgG2b (Figure 6C), and IgG2c (Figure 6D) antibodies than the Flu formulation (Figure 6) or the saline-solution inoculated animals (no anti-influenza antibodies detected; not shown). In addition, no statistically significant differences were found between the elicited antibody levels in caspase-1/11 wt and ko mice inoculated with the same formulations (Figure 6A,B,D), with the exception of IgG2b, where antibody levels elicited by IQB90-Flu were higher in wt than in ko mice (Figure 6C). We also assessed the ratio of IgG2c/IgG1 antibody levels in vaccinated C57Bl/6 mice. As shown in Figure 6E, IQB90-Flu was able to trigger a higher IgG2c/IgG1 ratio than the unadjuvanted formulation in an inflammasome independent-manner. This result suggests that IQB90-Flu is able to trigger a balanced Th1/Th2 response in C57Bl/6 mice, while IQB90-Flu vaccinated BALB/c mice elicited a more prominent polarization towards Th1 response (Figure 4B).

In order to further characterize the humoral immune response triggered by these nanoparticle formulations, we measured the number of specific ASCs in splenocytes of inoculated animals. At day 28, two weeks after boostering, splenocytes were obtained aseptically, and anti-influenza IgG ASC were assessed by ELISpot as explained Section 2. As shown in Figure 6F, no statistically significant differences were found between the wt and ko inoculated animals (Figure 6F). However, animals inoculated with IQB90-Flu deployed more influenza IgG-ASC than those inoculated with the unadjuvanted commercial vaccine (Figure 6F). This result is in line with the antibody levels found in sera and was explained previously. However, in further studies, it would be interesting to study the memory cells that can be recruited by animals inoculated with the different formulations once the booster effect has vanished.

### 3.7. Casp-1/11 ko Mice Vaccinated with IQB90-Flu Developed a Lower Delayed-Type Hypersensitivity (DTH) Response Than wt Mice

The DTH assay has, for a long time, been used to provide an additional measure of CMI towards a specific antigen, and it was assessed for the wt and casp1/11 ko mice as described in Section 2. Figure 6G shows both footpads of a wt mouse vaccinated with IQB90 24 h post i/d inoculation and the elicited DTH reaction. Animals vaccinated with Flu did not show any DTH reaction (Figure 6H,I), like the two mock vaccinated groups (not shown). However, animals vaccinated with IQB90-Flu did show a strong DTH response at 24 h, both in wt and casp-1/11 ko mice (Figure 6H). Forty-eight hours post i/d injection, ko mice showed a diminished DTH reaction (*p* < 0.05) in comparison with that elicited in the wt group (Figure 6I). This suggests that the CMI response triggered by the IQB90-Flu relies, at least in part, on a mechanism involving the Casp-1/11 pathway. Previously, Wilson and co-workers reported that ISCOMATRIX^TM^ nanoparticles (formulated with a *Q. saponaria* purified saponin fraction, CSL) activate the NLRP3 inflammasome, caspase-1 being a key player during such activation [41]. Our results presented herein are in line with previous reports by Detienne and colleagues when assessing the immune response triggered by QS-21 incorporated into cholesterol-containing liposomes: caspase-1/11 was found to be involved in the CMI response but not in the humoral immune response triggered by the saponin QS-21 [43]. Although triggering CMI responses is crucial to tackling intracellular infections, including those caused by the influenza virus, most currently used adjuvants (such as aluminum salts) fail to induce this kind of response. As such, this constitutes a major challenge in the development of new vaccine adjuvants [44]. Commercial saponin-based adjuvants and ISCOM nanoparticles have proven to induce this kind of immune response [8] as well as *Q. brasiliensis* saponins-based adjuvants [13], although their mechanism of action remains to be fully elucidated. Systems analysis and omics (such as proteomics, lipidomics, transcriptomics, systems serology, and metabolomics) have enhanced the current understanding of vaccine-adjuvants mechanisms over the last years [6]. These have become crucial in order to improve the current understanding of the mechanism of action of these adjuvants. Nonetheless, the induction of a CMI response showed herein by IQB90-Flu constitutes a promising result that demonstrates that it is worth continuing to study and deepen our knowledge to understand this mechanism.

## 4. Concluding Remarks and Perspectives

Saponin-based adjuvants were actively used in the past for veterinary vaccines, although for toxicity reasons, they were not used in human vaccines. In recent years, technological advances in saponin-fraction purification and nanoparticle formulations for vaccines were able to overcome the toxicity promoted by these molecules, and saponins are gaining attention for use in human health, as remarkable evidence has been gathered regarding their immune-stimulating potential. Importantly, two vaccines containing in the formulation a purified saponin from *Q. saponaria* have been recently approved for humans [9]. However, despite the relentless efforts made in order to improve the yield or find an alternative source for obtaining the saponin fractions from the *Q. saponaria* bark tree, scarcity, ecological threats to trees, yield of saponins obtained from raw material, and lack of reproducibility among batches are still challenges that need to be addressed [9,45]. Considering the similarities but also the comparative advantages of this saponin regarding commercially available *Q. saponaria* saponins, it is of utmost importance to keep working towards the understanding of their mechanism of action and adjuvant effect. In this sense, leaf-extracted *Q. brasiliensis* saponins arise as an alternative and renewable source of saponins.

Regarding the scaling-up of these novel adjuvants, it is important to highlight that the QB-90 fraction used here was obtained following exhaustive standardized procedures already reported [24]. In addition, physicochemical characterization methods were described and validated in order to analyze and quantify the QB-90 saponins fractions obtained [19]. In particular, using LC-MS [46,47], MALDI-TOF [18], and DI-ESI-TOF [17] for the characterization of *Q. brasiliensis* saponins have been previously described as valuable, fast, and cheap tools to analyze saponins fractions.

Although QB-90 saponin fraction is a natural product and, as such, presents an inherent variability, it is important to bear in mind that this drawback has already been overcome for *Q. saponaria* saponins, which are also from natural origin and very similar chemically and, thus, similar processes could be implemented for the large-scale obtainment of *Q. brasiliensis* saponins. Biotechnological methods, standard procedures, and GLP conditions are very powerful tools that have made it possible to systematize the extraction of natural complex products. In terms of ecological impact, *Q. saponaria* saponins require deforestation, as they are obtained from the bark of the trees, causing a large negative impact on the ecosystem. *Q. brasiliensis* saponins, however, are obtained from the leaves of the trees, so their fell is not required, and the forest can regenerate more quickly, which is a major advantage of using this novel adjuvant.

## 5. Conclusions

Influenza vaccines are key players in reducing seasonal and pandemic influenza burden and severity in a population. Nonetheless, many efforts are being deployed to achieve higher levels of protection, such as for the elderly, for which current commercially-available vaccines are not effective enough [5]. In this matter, the addition of *Q. brasiliensis* saponin-based adjuvants to influenza vaccines, in particular the ISCOM-like nanoparticles formulated with QB-90 saponins, arise as a possibility, as they have proven to increase the potency of the vaccine and to elicit stronger cellular and humoral immune responses than the unadjuvanted influenza vaccine as assessed in this work. In addition, a dose-sparing effect conferred by adjuvants is crucial in order to reduce the amount of antigen needed within vaccines to overcome antigen scarcity, especially when vaccines are highly required, for instance during a pandemic. IQB90-Flu and has proven to confer a ten-fold dose-sparing effect and, thus, could be useful for circumventing this issue.

Many efforts have been deployed in order to overcome the low-yield process and the ecological impact of saponin extraction, which include mass production of *Quijalla* forests [48] and the chemical synthesis of saponins [49]. Although to date it is not possible to obtain great amounts of saponins by either of these methods, their development is of the utmost importance in order to achieve a definitive sustainable source of saponins, which have proven to be remarkable adjuvants for use in vaccines that could address unmet medical needs such as low immunological response to influenza vaccines by specific populations or preparedness for the next influenza vaccine.

For all these reasons, the results obtained herein with *Q. brasiliensis*-based adjuvants encourage further investigations to move closer to clinical trials in the near future as an alternative to currently available commercial saponin-based adjuvants.

## Data Availability

Not applicable.

## References

[1] World Health Organisation (2019). Global Influenza Strategy Summary 2019–2030 Influenza. Glob. Influ. Strateg. 2019–2030 Influ..

[2] Paules C.I., Fauci A.S. (2019). Influenza Vaccines: Good, But We Can Do Better. J. Infect. Dis..

[3] Hobson D., Curry R.L., Beare A.S., Ward-Gardner A. (1972). The role of serum haemagglutination-inhibiting antibody in protection against challenge infection with influenza A2 and B viruses. J. Hyg. Lond..

[4] Seibert C.W., Rahmat S., Krause J.C., Eggink D., Albrecht R.A., Goff P.H., Krammer F., Duty J.A., Bouvier N.M., Garcia-Sastre A. (2013). Recombinant IgA Is Sufficient To Prevent Influenza Virus Transmission in Guinea Pigs. J. Virol..

[5] Tregoning J.S., Russell R.F., Kinnear E. (2018). Adjuvanted influenza vaccines. Hum. Vaccines Immunother..

[6] O’Hagan D.T., Lodaya R.N., Lofano G. (2020). The continued advance of vaccine adjuvants–‘we can work it out’. Semin. Immunol..

[7] Dalsgaard K. (1974). Saponin Adjuvants-Isolation of Substance/tom Quillaja saponaria Molina with Adjuvant Activity in Foot-and-Mouth Disease Vaccine. Arch. Gesamte Virusforsch..

[8] Magedans Y.V.S., Yendo A.C.A., de Costa F., Gosmann G., Arthur G. (2019). Foamy matters: An update on Quillaja saponins and their use as immunoadjuvants. Future Med. Chem..

[9] Lacaille-Dubois M.A. (2019). Updated insights into the mechanism of action and clinical profile of the immunoadjuvant QS-21: A review. Phytomedicine.

[10] Keech C., Albert G., Cho I., Robertson A., Reed P., Neal S., Plested J.S., Zhu M., Clark S.C., Zhou H. (2020). Phase 1–2 Trial of a SARS-CoV-2 Recombinant Spike Protein Nanoparticle Vaccine. N. Engl. J. Med..

[11] Heath P.T., Galiza E.P., Baxter D.N., Boffito M., Browne D., Burns F., Chadwick D.R., Clark R., Cosgrove C., Galloway J. (2021). Safety and Efficacy of NVX-CoV2373 Covid-19 Vaccine. N. Engl. J. Med..

[12] Morein B., Sundquist B., Höglund S., Dalsgaard K., Osterhaus A. (1984). Iscom, a novel structure for antigenic presentation of membrane proteins from enveloped viruses. Nature.

[13] Cibulski S.P., Silveira F., Mourglia-Ettlin G., Teixeira T.F., dos Santos H.F., Yendo A.C., de Costa F., Fett-Neto A.G., Gosmann G., Roehe P.M. (2016). Quillaja brasiliensis saponins induce robust humoral and cellular responses in a bovine viral diarrhea virus vaccine in mice. Comp. Immunol. Microbiol. Infect. Dis..

[14] De Costa F., Yendo A.C.A., Cibulski S.P., Fleck J.D., Roehe P.M., Spilki F.R., Gosmann G., Fett-Neto A.G. (2014). Alternative inactivated poliovirus vaccines adjuvanted with Quillaja brasiliensis or Quil-A saponins are equally effective in inducing specific immune responses. PLoS ONE.

[15] Yendo A.C.A., de Costa F., Cibulski S.P., Teixeira T.F., Colling L.C., Mastrogiovanni M., Soule S., Roehe P.M., Gosmann G., Ferreira F.A. (2016). A rabies vaccine adjuvanted with saponins from leaves of the soap tree (Quillaja brasiliensis) induces specific immune responses and protects against lethal challenge. Vaccine.

[16] Silveira F., Cibulski S.P., Varela A.P., Marqu??s J.M., Chabalgoity A., de Costa F., Yendo A.C.A., Gosmann G., Roehe P.M., Fern??ndez C. (2011). Quillaja brasiliensis saponins are less toxic than Quil A and have similar properties when used as an adjuvant for a viral antigen preparation. Vaccine.

[17] Cibulski S., Teixeira T.F., Varela A.P.M., de Lima M.F., Casanova G., Nascimento Y.M., Fechine Tavares J., da Silva M.S., Sesterheim P., Souza D.O. (2021). IMXQB-80: A Quillaja brasiliensis saponin-based nanoadjuvant enhances Zika virus specific immune responses in mice. Vaccine.

[18] Cibulski S., Rivera-Patron M., Suárez N., Pirez M., Rossi S., Yendo A.C., de Costa F., Gosmann G., Fett-Neto A., Roehe P.M. (2018). Leaf saponins of Quillaja brasiliensis enhance long-term specific immune responses and promote dose-sparing effect in BVDV experimental vaccines. Vaccine.

[19] Fleck J.D., De Costa F., Yendo A.C.A., Segalin J., Dalla Costa T.C.T., Fett-Neto A.G., Gosmann G. (2013). Determination of new immunoadjuvant saponin named QB-90, and analysis of its organ-specific distribution in Quillaja brasiliensis by HPLC. Nat. Prod. Res..

[20] Luebert F. (2014). Taxonomy and distribution of the genus Quillaja Molina (Quillajaceae). Feddes Repert..

[21] Fleck J.D., Kauffmann C., Spilki F., Lencina C.L., Roehe P.M., Gosmann G. (2006). Adjuvant activity of Quillaja brasiliensis saponins on the immune responses to bovine herpesvirus type 1 in mice. Vaccine.

[22] Cibulski S.P., Mourglia-Ettlin G., Teixeira T.F., Quirici L., Roehe P.M., Ferreira F., Silveira F. (2016). Novel ISCOMs from Quillaja brasiliensis saponins induce mucosal and systemic antibody production, T-cell responses and improved antigen uptake. Vaccine.

[23] Cibulski S.P., Rivera-Patron M., Mourglia-Ettlin G., Casaravilla C., Yendo A.C., Fett-Neto A.G., Chabalgoity J.A., Moreno M., Roehe P.M., Silveira F. (2018). Quillaja brasiliensis saponin-based nanoparticulate adjuvants are capable of triggering early immune responses. Sci. Rep..

[24] Yendo A., de Costa F., Kauffmann C., Fleck J., Gosmann G., Fett-Neto A., Fox C.B. (2017). Purification of an Immunoadjuvant Saponin Fraction from Quillaja brasiliensis Leaves by Reversed-Phase Silica Gel Chromatography. Vaccine Adjuvants: Methods and Protocols.

[25] Lendemans D.G., Myschik J., Hook S., Rades T. (2005). Immuno-stimulating complexes prepared by ethanol injection. J. Pharm. Pharmacol..

[26] Lv J., Wang D., Hua Y.H., Pei S.J., Wang J., Hu W.W., Wang X.L., Jia N., Jiang Q.S. (2014). Pulmonary immune responses to 2009 pandemic influenza A (H1N1) virus in mice. BMC Infect. Dis..

[27] Sun H.X., Xie Y., Ye Y.P. (2009). ISCOMs and ISCOMATRIX^TM^. Vaccine.

[28] McKenzie A., Watt M., Gittleson C. (2010). ISCOMATRIX vaccines: Safety in human clinical studies. Hum. Vaccin..

[29] Cebon J.S., Gore M., Thompson J.F., Davis I.D., McArthur G.A., Walpole E., Smithers M., Cerundolo V., Dunbar P.R., MacGregor D. (2020). Results of a randomized, double-blind phase II clinical trial of NY-ESO-1 vaccine with ISCOMATRIX adjuvant versus ISCOMATRIX alone in participants with high-risk resected melanoma. J. Immunother. Cancer.

[30] Bigaeva E., van Doorn E., Liu H., Hak E. (2016). Meta-Analysis on Randomized Controlled Trials of Vaccines with QS-21 or ISCOMATRIX Adjuvant: Safety and Tolerability. PLoS ONE.

[31] Holmgren J., Czerkinsky C. (2005). Mucosal immunity and vaccines. Nat. Med..

[32] De Magistris M.T. (2006). Mucosal delivery of vaccine antigens and its advantages in pediatrics. Adv. Drug Deliv. Rev..

[33] Mitragotri S. (2005). Immunization without needles. Nat. Rev. Immunol..

[34] De Temmerman M.L., Rejman J., Demeester J., Irvine D.J., Gander B., De Smedt S.C. (2011). Particulate vaccines: On the quest for optimal delivery and immune response. Drug Discov. Today.

[35] Kunda N.K., Somavarapu S., Gordon S.B., Hutcheon G.A., Saleem I.Y. (2013). Nanocarriers targeting dendritic cells for pulmonary vaccine delivery. Pharm. Res..

[36] Sjölander S., Drane D., Davis R., Beezum L., Pearse M., Cox J. (2001). Intranasal immunisation with influenza-ISCOM induces strong mucosal as well as systemic antibody and cytotoxic T-lymphocyte responses. Vaccine.

[37] Tomar J., Patil H.P., Bracho G., Tonnis W.F., Frijlink H.W., Petrovsky N., Vanbever R., Huckriede A., Hinrichs W.L.J. (2018). Advax augments B and T cell responses upon influenza vaccination via the respiratory tract and enables complete protection of mice against lethal influenza virus challenge. J. Control Release.

[38] Podda A. (2001). The adjuvanted influenza vaccines with novel adjuvants: Experience with the MF59-adjuvanted vaccine. Vaccine.

[39] Krammer F., Smith G.J.D., Fouchier R.A.M., Peiris M., Kedzierska K., Doherty P.C., Palese P., Shaw M.L., Treanor J., Webster R.G. (2018). Influenza. Nat. Rev. Dis. Prim..

[40] Reber A., Katz J. (2013). Immunological assessment of influenza vaccines and immune correlates of protection. Expert Rev. Vaccines.

[41] Wilson N.S., Duewell P., Yang B., Li Y., Marsters S., Koernig S., Latz E., Maraskovsky E., Morelli A.B., Schnurr M. (2014). Inflammasome-Dependent and -Independent IL-18 Production Mediates Immunity to the ISCOMATRIX Adjuvant. J. Immunol..

[42] Li H., Willingham S.B., Ting J.P.-Y., Re F. (2008). Cutting Edge: Inflammasome Activation by Alum and Alum’s Adjuvant Effect Are Mediated by NLRP3. J. Immunol..

[43] Detienne S., Welsby I., Collignon C., Wouters S., Coccia M., Delhaye S., Van Maele L., Thomas S., Swertvaegher M., Detavernier A. (2016). Central Role of CD169(+) Lymph Node Resident Macrophages in the Adjuvanticity of the QS-21 Component of AS01. Sci. Rep..

[44] Knudsen N.P.H., Olsen A., Buonsanti C., Follmann F., Zhang Y., Coler R.N., Fox C.B., Meinke A., D’Oro U., Casini D. (2016). Different human vaccine adjuvants promote distinct antigen-independent immunological signatures tailored to different pathogens. Sci. Rep..

[45] Fernández-Tejada A., Chea E.K., George C., Gardner J.R., Livingston P.O., Ragupathi G., Tan D.S., Gin D.Y. (2014). Design, synthesis, and immunologic evaluation of vaccine adjuvant conjugates based on QS-21 and tucaresol. Bioorganic Med. Chem..

[46] Wallace F., Bennadji Z., Ferreira F., Olivaro C. (2019). Structural characterisation of new immunoadjuvant saponins from leaves and the first study of saponins from the bark of Quillaja brasiliensis by liquid chromatography electrospray ionisation ion trap mass spectrometry. Phytochem. Anal..

[47] Wallace F., Bennadji Z., Ferreira F., Olivaro C. (2017). Analysis of an immunoadjuvant saponin fraction from Quillaja brasiliensis leaves by electrospray ionization ion trap multiple-stage mass spectrometry. Phytochem. Lett..

[48] Fleck J.D., Schwwambach J., Almeida M.E., Yendo A.C.A., de Costa F., Gossmann G., Fett-Neto A.G. (2009). Immunoadjuvant Saponin Production in Seedlings and Micropropagated Plants of Quillaja brasiliensis. Soc. Vitr. Biol..

[49] Fernández-Tejada A., Tan D.S., Gin D.Y. (2016). Development of Improved Vaccine Adjuvants Based on the Saponin Natural Product QS-21 through Chemical Synthesis. Acc. Chem. Res..

